# Reproductive rights for women with chronic kidney disease. Unfortunately, we are still in our infancy

**DOI:** 10.1590/2175-8239-JBN-2023-E008en

**Published:** 2023-04-17

**Authors:** Claudio Luders

**Affiliations:** 1 Hospital Sírio-Libanês, Centro de Nefrologia e Diálise, São Paulo, SP, Brazil.

There was a time in the history of nephrology when the possibility of a woman with chronic kidney disease (CKD) becoming pregnant was viewed with great distress. In 1975, an editorial published in The Lancet translated this sentiment into words: “Children of women with renal disease used to be born dangerously or not at all – not at all, if their doctors had their way”^1^. Despite the overall paternalistic stance physicians would take on the issue by advising against and even prohibiting pregnancy, many women did, nonetheless, become pregnant. The qualitative study published by Schützer et al. helps to understand the dynamics of the complex interrelationships between CKD and pregnancy from the point of view of women with the disease^2^. Although the experience of having CKD and becoming pregnant is utterly personal, qualitative studies can provide a more holistic view of these cases and improve the care provided to patients. Regardless of country, patients with CKD, on dialysis or not, have very similar experiences^3^. We followed more than 60 patients seen at the Hospital das Clinicas of the School of Medicine of the University of São Paulo who became pregnant while on dialysis^4^. The commonalities they shared included the fact that none used contraceptives or received information about the possibility of becoming pregnant while on dialysis; all believed that they were unable to get pregnant due to CKD; and none talked to their nephrologists about these issues. Lack of information about reproductive health for individuals with CKD appears frequently in qualitative reports. Between 33% and 50% of pregnancies in transplant patients were unplanned. Data from dialysis centers in the United States show that, although 50% of the patients were sexually active, only 36% used contraceptives and only 13% had discussed reproductive health with their nephrologists^5^.

Reproductive rights are understood as the ability women have to decide when and if they want to become pregnant. Motherhood should be a conscious choice, not something forced upon or denied to someone. Reproductive health and rights are part of the UN’s global human rights agenda^6^. How can patients make conscious choices if they are not informed about the possibilities and risks – for themselves and the children they carry – of becoming pregnant while with CKD? Nephrologists are the only physicians many patients on dialysis have regular access to. Unfortunately, nephrologists have not been trained to address topics such as contraception, sexual health, pregnancy, fertility, menstrual disorders, assisted reproduction, and menopause. This is not a problem unique to Brazil, but a concern that affects nephrologists worldwide^7^. In a survey conducted in Canada with 154 nephrologists, the authors found that more than 65% of the participants did not feel confident about providing advice about or managing specific problems related to women’s health, mostly due to lack of training during medical residency and/or specialization courses in nephrology. In addition, 90% of the interviewed physicians reported that they missed interdisciplinary seminars and ongoing education programs covering this area of knowledge^8^.

We need to be clear about the importance of women’s reproductive health. Between 3% and 6% of women of childbearing age have CKD, with 3.3% of all pregnancies occurring in patients with different stages of CKD. This is a growing trend worldwide, since women are getting pregnant at a later stage in their lives and the prevalence of obesity has increased in this population^5,8^. The prevalence of advanced kidney disease is 30% higher in women than in men, and renal complications during pregnancy certainly contribute to a higher risk^9^. Despite the higher prevalence of severe forms of CKD in women, fewer woman are prescribed dialysis and transplantation than men. Women on dialysis have the same mortality as men, thus reversing the advantage of lower mortality compared to men in different age groups. Pregnant women with CKD have a 10-fold increased risk of preeclampsia, a 5-fold increased risk of premature birth, and a 3-fold increased risk of having a cesarean delivery. Kidney disease in all stages may progress due to pregnancy. Progression can be mild for individuals with early-stage CKD, especially patients with proteinuria lower than 1.0 g/24 h, and severe for patients with advanced CKD with or without proteinuria^5^.

We are still in our infancy when it comes to health care and reproductive rights for women with CKD. However, the amount of knowledge accumulated in recent decades has allowed progress to begin. We need to engage medical residency committees and medical societies, and start discussion forums to broaden the debate and address the issue at hand. Interdisciplinary programs involving obstetricians and nephrologists, aiming to increase the quality and scope of the training of future residents, are of fundamental importance. Discussions about contraception, menstrual disorders, fertility, pregnancy, and menopause should be part of the everyday conversation between nephrologists and their patients, and a fundamental element in patient support and in the improvement of care and quality of life of women with CKD.
